# Genomic Analysis of Endophytic *Bacillus cereus* T4S and Its Plant Growth-Promoting Traits

**DOI:** 10.3390/plants10091776

**Published:** 2021-08-26

**Authors:** Bartholomew Saanu Adeleke, Ayansina Segun Ayangbenro, Olubukola Oluranti Babalola

**Affiliations:** Food Security and Safety Niche Area, Faculty of Natural and Agricultural Sciences, North-West University, Private Bag X2046, Mmabatho 2735, South Africa

**Keywords:** bacterial endophytes, greenhouse experiment, Illumina sequencing, oilseed crop, plant growth promotion, secondary metabolite genes

## Abstract

Insights into plant endophytic microbes and their exploration in agriculture have provided opportunities for sustainable plant health and food safety. Notable endophytic *Bacillus* species with plant growth-promoting traits have been documented; nevertheless, information on genome analysis of *B. cereus* associated with the sunflower in South Africa has not been studied. Therefore, we present whole-genome sequence of agriculturally important *B. cereus* strain T4S isolated from sunflower plants. The NextSeq Illumina sequencing yielded 7,255,762 bp sequence reads, 151 bp average read length, 5,945,881 bp genome size, 56 tRNA, 63 rRNA, and G + C content of 34.8%. The phylogeny analysis of strain T4S was similar to *B. cereus* NJ-W. Secondary metabolites, such as petrobactin, bacillibactin, bacitracin, molybdenum factor, zwittermicin, and fengycin underlining bacterial biocontrol efficacy against phytopathogens were found in the T4S genome. The predicted novel genes in the bacterial genome mediating the complex metabolic pathways can provide a genetic basis in understanding endosphere biology and their multiple functions thereof in crop improvement. Interestingly, seed and root inoculation with strain T4S contributed to sunflower yield under greenhouse experiments. Hence, the detection of notable genes specific for plant growth promotion as validated under in vitro screening, promisingly, suggests the relevance of strain T4S in agricultural biotechnology.

## 1. Introduction

In recent times, agricultural sustainability to maximally meet the nutritional food demand of the growing population needs a boost, more importantly, to avert future food scarcity and insecurity, and hunger. Notably, one of the promising measures is through agricultural intensification and research innovations into biotechnological functions of agriculturally important microbes inhabiting the endosphere of crops and how they can be explored as bioinoculants on a commercial scale [1,2].

Agricultural practices such as tillage, crop rotation, and fertilizer application contribute to food production, reduce pest re-insurgence, and plant-pathogen attack [3]. This has also ensured the availability of quantity and quality of food products for human beings [4]. However, the incessant use of agrochemicals by farmers to recover the essential nutrient loss in the soil for crop improvement has continually harmed the environment [5,6]. It has been postulated that indiscriminate or continuous use of agrochemicals does not have linkage to the quantity of food produced per annum, instead, it leads to negative environmental stimuli and consequences, particularly water contamination and pollution problems [7].

To mitigate these threats, employing best and acceptable practices to reduce the rate of chemical fertilization to enhance crop production remains fundamental to sustainable agriculture. These measures include harnessing endophytic bacteria as invaluable resources in exchange for agrochemicals to improve plant resistance against biotic and abiotic stresses [8]. Conversely, price instability, scarcity, and limited supply of chemical fertilizers due to the energy crisis, political, economic, and natural disasters can influence the rate of food production. In ecologically threatened regions, the plant is faced with a lot of stress adaptors, thus instigating their internal and physiological cellular responses [9]. Nevertheless, the recruitment of endophytic microbes as biological adjuvants to support plant growth in harsh or drought-prone areas can stimulate adaptive immune responses in plants [10].

Furthermore, employing endophytic bacteria as bio-factory in the synthesis of certain secondary metabolites can enhance their efficacy in agricultural biotechnology and implementation [11]. Endophytes are microorganisms that inhabit the internal tissue of the host plants without causing disease symptoms [12]. The mechanistic effects of these bacteria on plant growth-promotion can be direct or indirect. Significantly, plant growth-promoting endophytes (PGPE) support plants in diverse ways, mainly in the induction of systemic resistance, synthesis of phytohormones, antibiosis on phytopathogens, and competition for available nutrient and water in an ecological niche [13].

Based on the dominance of the genera *Bacillus* in different environments, their contributions to enhancing plant growth cannot be overemphasized. Additionally, their singularly or combined inoculation has shown significant effects on crop (wheat) yield compared to the un-inoculated [7,14]. The improvement in plant biomass and crop yields, such as rice, *Arabidopsis*, wheat, maize, sorghum, and soybean upon inoculation with PGPE as demonstrated, has enhanced their productivity [7,15]. Igiehon et al. [16] have reported an inconsistent and less effect of a single bacterial application on experimental plants. Nevertheless, co-inoculation of non-antagonistic bacteria is found significant, contributing to plant performance in terms of quality and quantity than single inoculation. Notwithstanding, Subramanian et al. [17] and Ríos-Ruiz et al. [18] have reported an improvement in the yield of soybean co-inoculated with endophytic *Bradyrhizobium japonicum* and *Bacillus megaterium* and rice yields inoculated with endophytic *Burkholderia ubonensis, Citrobacter bitternis,* and *Burkholderia vietnamiensis*, respectively.

The group of bacterial species in the genus *Bacillus* can be found in various ecosystems, based on the endospore attributes that make them persist in extreme environments [19]. The multiple functional attributes of endophytic bacteria in agriculture can underline their potential based on the expression of novel genes in the bacterial genome that is involved in various metabolic pathways. For example, sulfur metabolism, phytohormone, exopolysaccharide, siderophore synthesis, enzyme biosynthesis, tryptophan metabolism, nitrogen fixation, and photosynthesis [20].

The discovery of vital genes in *Bacillus* isolated from sunflower can make them suitable candidates for various biotechnological applications. Significantly, bacterial inoculation of economically relevant plants, such as sunflower, can contribute to growth and oil production. Sunflower is classified as one of the leading oilseed crops after soybean, safflower, and rapeseed, especially in South Africa [4]. Despite the economic importance of sunflower and the identification of *B. cereus* from other plants [14], little information is available on the genome analysis of *B. cereus* associated with the root of sunflower in South Africa. *B. cereus* is a Gram-positive, spore-forming, rod-like bacterium belonging to the *Bacillus* genus. The whole-genome sequencing of *B. cereus* isolated from the rhizosphere and endosphere of plants has revealed notable genes in their genome, more importantly, plant growth-promoting genes [21,22]. In addition, Hong et al. [23] have reported the complete genome sequence of endophytic bacterium *B. cereus* strain PgBE311 isolated from *Panax ginseng*. However, reports on the *B. cereus* isolated from the sunflower are less documented, thus suggesting its exploration and application in improving crop yield. Sunflower yield can be improved depending on the efficacy of the copious associated bacterial endophytes upon inoculation under experimental conditions. Therefore, we report the isolation, cultured-based screening of plant growth-promoting traits, and genomic characterization of endophytic bacterium *B. cereus* T4S isolated from sunflower root endosphere, and its effect on sunflower yield under greenhouse experiment.

## 2. Results

### 2.1. Biochemical and Cultural Features

**Figure 1:**
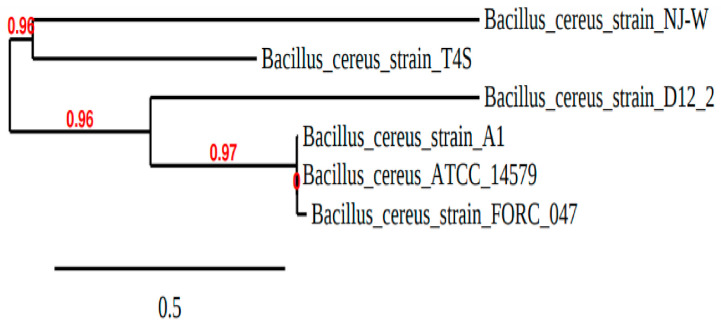
Phylogeny of the genome sequence data of *B. cereus* T4S. **Legend**: Scheme 0. means 96% difference between two bacterial sequences.

The biochemical and cultural characterization of *B. cereus* T4S is presented in Table S1. The results indicated that *B. cereus* T4S is rod-like, catalase-positive, and Gram-positive. The strain T4S assimilates all the tested sugars and exhibited a positive reaction to citrate, oxidase, nitrate, casein, and starch hydrolysis. Furthermore, strain T4S grows between pH ranging from 4 to 10, temperature from 25 to 45 °C, and 5.5% normal saline. The results of plant growth-promoting tests were high, with a siderophore value of 87.30%, phosphate content of 30.43 µg/mL, and IAA of 11.28 µg/mL, respectively (Table 1). Bacterial endophytes exhibited significant reactions for protease, xylanase, mannanase, and cellulase production, except amylase during enzyme plate assay. The phylogeny of the genome sequence of *B. cereus* strain T4S is presented in Figure 1.

### 2.2. Whole Genome Sequencing Analysis

**Figure 2:**
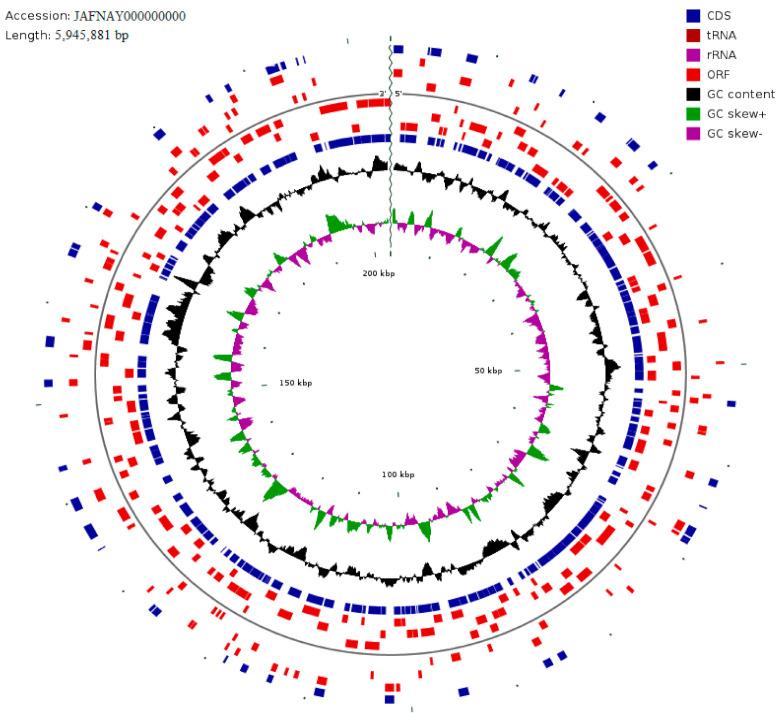
Circular genome visualization of the *Bacillus cereus* strain T4S. Each color from the external to internal circle depicts green (GC skew +), and red (ORF). The ring black coloration (GC content) at the peak indicated higher or lower values than average GC content. The GC Skew (−/+) in purple/green peaks in/outside the circle indicated values greater or smaller than 1. The GC Skew is calculated as G−C/G + C.

The WGS analysis of *B. cereus* T4S yielded a sequence read count of 7,255,762, a genome size of 5,945,881 bp, total bases of 109,562,062, and G + C content of 34.8%. The read length mean was 151 bp while *L*_50_ and *N*_50_ values were 32 and 65,078 bp, respectively. The number of contigs and subsystems was 198 and 341, respectively. The genome analysis revealed 6277 coding sequences and 63 RNAs. The circular genome visualization of *B. cereus* strain T4S is represented in Figure 2. The subsystem statistics show 27 subsystem feature counts of the coding protein into functional groups based on the annotated genome classification by SEED in RAST. The functional basis of 5159 protein-coding genes (PCG) was assessed from the KEGG database and RAST online server. The 1961 genes annotated by SEED viewer (version 2.0) were grouped into molecular function, cellular components, and biological processes. The topmost five groups were protein metabolism (155 genes), vitamins, prosthetic groups, pigments (161 genes), cofactor, amino acids and derivatives (377 genes), carbohydrates (271 genes), and nucleosides and nucleotides (119 genes).

The PCG from the genome of strain T4S revealed various genes involved in biocontrol, biomolecule transport and degradation, metabolic pathway, and synthesis of cellular components. Moreover, their locus tag, specific gene type, and gene product (Table 2, Table 3, Table 4 and Table 5, Tables S2–S13). In Table S2, various secretion systems genes are highlighted. It was revealed that *B. cereus* T4S harbors nitrogen fixation genes (*nif*U and *nif*3-like) and other genes involved in ammonia and urea transport, nitrogen metabolism, nitrogen regulation, and nitrate reduction pathway (Table 2).

The presence of PCG involved in the degradation of phosphonates such as *phn*CE and *isp*E, and phosphate transport (*pst*ABCS, *pho*UH, *glt*P, and *ugp*C) in *B. cereus* T4S is shown in Table 3. Similarly, the genes mediating sulfate transport were detected in the genome of *B. cereus* T4S (Table S3). *B. cereus* T4S possesses genes involved in iron transport and siderophore production, which include, *fbp*A, *feo*AB, and *fet*B (Table 4). Several other genes responsible for flagellar biosynthesis (*flg*BDEGK, *flh*ABG, *fli*CEFKLMNPQRSY, and *mot*AB), chemotaxis (*che*VW), and biofilm formation (*efp, hfq,* and *crp*) were detected in *B. cereus* T4S (Table S4). Other genes that mediate lipopolysaccharide biosynthesis were also detected in strain T4S (Table S5). Genes involved in the degradation of superoxide anion radicals and hydrogen peroxide (*sod*A and *kat*B), as well as gene *ahp*C coding for peroxiredoxin, were detected in the genome of *B. cereus* T4S (Table S6). Other related genes involved in carbohydrate metabolism and transport, organic acid metabolism, amino acid degradation and transformation, opine and GABA transport and metabolism, lignin degradation and degradation against toxic peroxides, and modulation of plant hormones are presented in Table 5 and Tables S7–S12.

Subsequently, identifiable genes specific for biological control such as isoprene (*isp*G), colonization (*min*D), 2,3-butanediol (*ilv*ABCDEGN), and volatile degradation (*aco*AB) pathways were identified in the genome of *B. cereus* T4S (Table S13).

### 2.3. Predicted Secondary Metabolite Cluster Genes by antiSMASH

**Figure 3:**
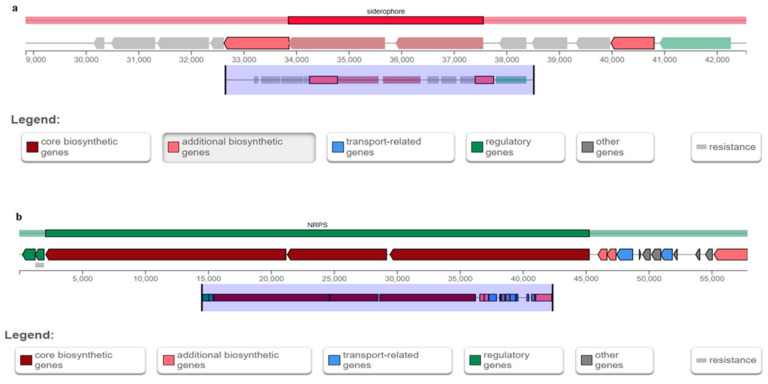
(**a**,**b**): Graphical representation of siderophore-and-nonribosomal peptide (NRPS)-encoding genes. These loci are predicted from the whole genome assembly of *B. cereus* T4S.

Table 6 shows the predicted secondary metabolite cluster genes from antiSMASH analysis of *B. cereus* T4S. Six notable secondary metabolite cluster genes detected include regulatory genes, other genes, additional biosynthetic genes, core biosynthetic genes, resistance genes, and transport-related genes (Figure 3a,b). *B. cereus* T4S exhibited 100% similarity for gene type siderophore and non-ribosomal peptides (NRPS), with the most similar known cluster for petrobactin and bacitracin (Table 6). The siderophore-and-NRPS-encoding genes in *B. cereus* T4S were found in the location between 28,853 and 42,560 nt. (total: 13,708 nt) as well as 1 and 57,852 nt. (total: 57,852 nt) of the entire genomic region.

### 2.4. Effect of Bacterial Inoculation on Sunflower Yield

**Figure 4:**
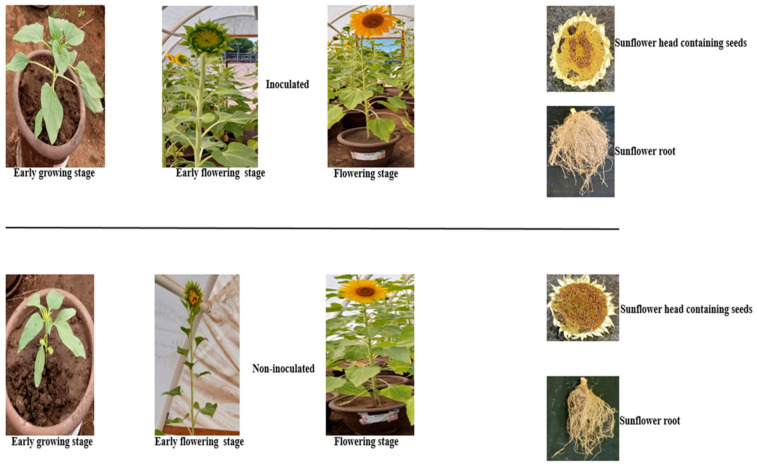
Inoculated and non-inoculated sunflower growth and yield parameters.

The resulting output from the greenhouse experiment is presented in Table 7. Inoculated sunflower with *B. cereus* T4S statistically was significantly different in the growth parameter compared to non-inoculated (Figure 4). Here, the results show higher yields in the parameters measured in the inoculated plant than un-inoculated, except for the seed dry weight with no significant difference. The physical and chemical analysis of the soil sample recorded high magnesium content, 642 (mg/kg), followed by potassium content, 399 (mg/kg), and the least value 0.10% of total nitrogen content (Table S14). The clay, sand, and silt values were 22.20%, 68.30%, and 7.92%, respectively.

## 3. Discussion

In recent times, the potential of microbial endophytes ranging from plant growth promotion to control of phytopathogens to ensure agriculture sustainably remains fundamental [24]. Significantly, the detection of multifunctional genes from the diverse bacterial community in the roots endosphere by whole genome sequence analysis with biotechnological prospecting has provided salient information on the metabolic activities and their agricultural importance [25]. Nevertheless, this approach has gainfully acquitted researchers with a motivational framework in the exploration of environmental microbes as a key bio-factory of various compounds, importantly for both present and future use. Biotechnologically, the accumulated information on less studied endophytic microbes in the sunflower endosphere would significantly necessitate more research, focusing on plant beneficial bacterial endophytes with multifaceted metabolic potential. To this premise and based on scanty information relating to the whole genome analysis of endophytic bacteria in the sunflower root endosphere (SRE), we present this research as a promising genomic overview into SRE biology.

The genomic sequence dataset of *B. cereus* strain T4S from the SRE was analyzed. The identified strain T4S potentially produces lytic enzymes, IAA, siderophores, and also, displayed the ability to solubilize phosphate. To corroborate this, research findings into plant growth-promoting activities of endophytic bacteria colonizing the root endosphere of sunflower based on their multifunctional traits could functionally ensure better crop yields [26].

The genomic insights into *B. cereus* T4S might perhaps unravel its diverse bioprospecting abilities, channeling appropriate specific mechanisms of action in the host plants. The abundance of exudates in the root environment can facilitate rhizobacteria infiltrating from the external root environment into the root endosphere and become endophytes [27]. Notably, exudate secretions from the plant roots can enhance bacterial colonization and establishment of plant-microbes equilibrium in the root endosphere, thus significantly improve plant performance [28]. Bacterial root colonization has been attributed to the presence of specific genes involved in biofilm formation, chemotaxis, and flagellation in their genome, thus enabling them to establish a bacterial community in the plant endosphere with multiple functions to promote plant growth under water-deficit stress. The phylogeny analysis of the genome sequence of *B. cereus* T4S revealed a close relatedness to *B. cereus* NJ-W. The positioning of taxonomic classification of bacteria can be resolved based on gene sequences.

The detection of several genes involved in protein secretion systems and TAT pathways is presented in this study. Peptidase could mediate the export of protein by cleaving the N-terminal of a signal peptide. Secretions of complex genes such as *sec*Y, *sec*E, *sec*D, *sec*G, and *sec*F in the cytoplasm of bacteria are known [29]. The presence of *Sec*A in most bacteria facilitates the binding of signal peptides as a driver in the translocation of protein through other gene channels, like *sec*YEG. Moreover, *sec*B in the cytoplasm could contribute to protein stability and secretion within the cell. Exhibition of oxidative gene encoding pyrroloquinoline quinone (PQQ)-dependent sugar dehydrogenase by strain T4S could be an indication of bacterial resistance or adaptation to reactive oxygen species (ROS) produced under a stress-induced environment in building strong defense mechanisms in the host plants [30,31].

The presence of nitrogen fixation genes, *nif*3-like and *nif*U by strain T4S revealed that they can participate effectively in biological nitrogen fixation through the conversion of dinitrogen gas into nitrate and ammonium within the plant tissues. The biological nitrogen fixation by *Bacillus* spp. in chicken-pea grown in nitrogen-deficient soil environment has been reported [32]. Recently, the identification of *nif*U in *P. aeruginosa* B8 isolated from the root of sugar with nitrogen fixation potential has been documented [25]. Phosphorus is either fixed or immobilized in the soil, thus limiting its usage by plants. Phosphorus is made available to plants by phosphate-solubilizing microorganisms, especially the endophytic type capable of synthesizing phosphatases and organic acids [11]. Different *Bacillus* strains such as *B. pumilis, B. subtilis, B. amyloliquenfaciens,* and *B. methylotrophicus* are regarded as phosphate solubilizers, and their use in soil management has enhanced phosphorus level in soil [33,34]. The screened genome of strain T4S uncovered genes involved in phosphonate degradation and phosphate transport pathways. Various genes involved in phosphate solubilization and transport include *phb*CEF, *isp*H, *pst*ABCS, *pho*U, *glt*P, and *ugp*C. The presence of *pst* encoding phosphate transport system permease protein as predicted can be accountable for an increase in phosphate uptake and bioavailability in a phosphate-limiting environment [30], thus contributing to plant growth. The role of *pst* in the phosphate transport pathway by *B. subtilis* MBI 600, *B. cereus* 905, and *Paenibacillus polymyxa* HK4 has been documented [22,29,35]. Notably, the detection of phosphate transport genes (*pst*ABCS) correlates with the finding of Zeng, Xie, Li, Gao, Xu, and Wang [22], who reported the same genes in the whole genome of plant growth-promoting *B. cereus* strain 905. Similarly, Singh et al. (2021) have reported the presence of phosphate transport genes (*pst*ABCS) in the genome of *P. aeruginosa* B18. Nevertheless, the authors have also reported *pho*BDHR, which was not detected in this study. In another study, the presence of phosphate transporter gene, *pho*RP in the genome of *B. cereus* was documented [22]. The presence of phosphonate degradation and phosphate transport genes in stain T4S could enhance the rate of phosphate utilization by the host plants.

The presence of sulfur transport genes in the genome of an endophytic bacterium can facilitate the degradation of sulfur-containing xenobiotic compounds. It also contributes to the sulfur acquisition ability of bacterial strain and possibly in the modulation of sulfur levels in plants and soils. The identification of sulfate transport genes (*sul*P, *cys*APUW, and *yln*A) in *B. megaterium* strain STB has been documented to enhance plant growth and mitigate stress in plants [31].

Plants tend to survive in iron-limiting soil due to the abundance of siderophore-producing microbes that ensure the acquisition and bioavailability of soluble iron for plants and microbial uptake [36]. Siderophore production can exert lethal effects on targeted phytopathogens due to the strong affinity of microbes to compete for iron in the soil [37]. Siderophore activity of strain T4S was profound. The genome revealed the presence of genes, such as *fep*A, *feo*A, *feo*B, and *fet*B that code for iron transport, thus suggesting their strong linkage for siderophore production. The expression of siderophore genes, namely, pyoverdine homologous genes, *pvd, fhu, mbt*H, *fpv*A, and *acr*AB from bacterial genera with potential plant growth promoting traits have been studied [38]. Douriet-Gámez et al. [39] have identified siderophore genes *dhb*ABCF from *Bacillus* sp. strain B25. The results here corroborate the study of Nascimento, Hernandez, Glick, and Rossi [30] who reported the presence of genes (*feo*AB) involved in iron transport in *Pantoea phytobeneficialis* strain MSR2. Furthermore, reports on several putative siderophore genes (*rha*ABCDEF) that somewhat mediate the synthesis of rhizobactin for the production of hydroxamate-like siderophore of *B. megaterium* STB1 and *Sinohizobium meliloti* 1021 are known [31]. Interestingly, active participation of *B. megaterium* STB1 in the siderophore transport pathway due to the presence of siderophore genes, *yfi*Z, *yfh*A, *yfi*Y, and *yus*V that regulate iron transport in *B. subtilis* have suggested their influence in competitive interactions with potential pathogens and enhancement of plant growth [40]. In this study, various genes involved in biofilm formation, chemotaxis, and flagellation were detected in the genome of an endophytic bacterium strain T4S. The presence of lipopolysaccharide (LPS) genes can enhance bacterial functions to firmly stabilize their outer membrane from desiccation. Interestingly, strain T4S harbors gene *glm*U encoding glucosamine-1-phosphate N-acetyltransferase involved in LPS, lipid A biosynthesis pathways. The presence of flagellar motor protein genes (*mot*AB) was in line with Nascimento, Hernández, Glick, and Rossi [31], who have earlier reported the same genes in the genome of *B. megaterium* STB1. The authors further documented several other genes that were involved in the synthesis of exopolysaccharides that enhances biofilm formation and adherence of bacteria to the plant root. Similarly, teichoic acid genes responsible for the biofilm formation and root attachment were documented [31].

One of the key attributes of endophytic bacteria is the ability to produce exopolysaccharides (EPS). The expression of EPS genes in the bacterial genome has contributed to plant resistance under drought conditions and the formation of biofilm that aids surface colonization [41]. EPS functions in biofilm production confer defensive mechanisms in response to ROS and plant protection against pathogens. The biofilm formation/production by endophytic microbes could enable plants to resist environmental stressors and firm colonization within the endosphere, thus suggesting their functions in plant-microbe interactions [30]. Furthermore, EPS synthesis enhanced bacterial affinity within the host plants for various biological functions [25]. Various genes such as *flg*BDFGK, *flh*ABE, *fli*CEHFKLMNPQRSY, and *mot*AB are found in the genome of strain T4S involved in the flagellar biosynthesis pathway. Protein coding gene *crp* that is primarily involved in biofilm production was also identified in strain T4S. The identification of specific genes involving in biofilm production from the genome of endophytic bacterial strain associated with the root endosphere is known [25,29]. Based on a previous study by Xu et al. [42], it can be inferred that *B. cereus* T4S might have potential as a biocontrol agent due to the presence of certain gene families which have been demonstrated to be responsible for the biocontrol efficacy in experiments performed in other host/microbe systems.

Carbon sources provide the required energy for bacterial metabolism. Endophytic bacterial strain T4S contains genes involved in carbohydrate metabolism and transport through dynamic pathways, which include, pentose phosphate pathway, EMP (Embden–Meyerhof–Parnas) glycolysis pathway, maltose, cellobiose, glucose, and ribose transport pathways, hexose metabolism pathway, GDP-mannose, N-acetyl-D-galactosamine and D-galactosamine, trehalose, myo-inositol, D-gluconate, L-lactate, L-lyxose, beta-glucosides, 2,3-diketo-L-gulonate-and-ascorbate, chitin, D-tagatose, and fructose degradation pathways, sugar transport, sugar acids transport, and trehalose-specific EIIBC component. The ability of bacterial endophytes to utilize complex carbohydrate compounds has significantly contributed to the establishment of plant-microbe interactions. Reports have shown that the amount of metabolizable sugar released from plant roots could mediate the colonization tendencies of bacterial in the root endosphere compartments and then contribute to crop yield [43,44]. Thus, the genomic sequencing of *B. cereus* T4S reveals the presence of gene families with previously reported beneficial roles in other crop-microbe associations [45], which suggests that *B. cereus* T4S may be a candidate in stimulating phytohormones in enhancing sunflower growth.

Owing to the genetic profiling of bacterial endophytes, they can participate in carbohydrate metabolic pathways and related carbon derivatives from the plant root exudates. The amount of carbohydrate content in plants can mediate sugar signal networking for cellular regulation and a shift in plant response to environmental stress adaptors [46]. Reports on the utilization of glucose, sucrose, maltose, and ribose as a carbon source by bacterial endophytes have been shown to have contributed to their metabolic activities [47]. Biotransformation of organic compounds for organic acids production could share a direct link for enhanced bacterial colonization with the host plants.

Production of notable genes involved in amino acid biodegradation and biotransformation could contribute to the functional attributes of bacteria in eco-niches. The presence of genes in the polyamine, amino acid opine, and 4-aminobutyrate (GABA) metabolism in strain T4S have suggested their ability to metabolize amino acid, thus contributing to microbial colonization for improved plant growth. Moreover, the presence of opines could be responsible for amino acid condensation. Opines are known as one of the chemical compounds produced by rhizobacteria that participate in plant-bacteria colonization, thus modulating root bacterial-nodulation processes. The protein-encoding myo-inositol and rhizopine metabolism (*iol*CDEG) involve in the degradation of these compounds suggested the importance of these compounds in bacterial colonization with plants. The role of GABA synthesis by bacterial species in synergistic interactions with plants for enhanced plant performance has been reported [30,48].

The possession of lignin degradation encoding genes, multicopper oxidase domain-containing protein has yielded other organic compounds such as alcohols, phenolic and toxic aldehydes, several intermediates-like alcohols (gallate and protocatechuate), and aromatic compounds, vanillate [30,49]. The presence of lignin-degrading genes and other plant components could significantly facilitate endophytic colonization.

The role of root-associated bacterial endophytes such as *Burkholderia, Bacillus, Pseudomonas* in plant growth promotion has been reported in recent times with a promising outlook in agriculture sustainably [25,50,51]. Nevertheless, information regarding the phytohormone production ability of bacterial endophytes from the sunflower root endosphere as revealed by whole-genome analysis is less studied. Plant hormone synthesis is a major feature that distinguishes agriculturally important bacteria from one and another [52]. The detection of *acd*A could serve as a precursor in lowering ethylene levels in plants. Deamination of 1-aminocyclopropane-1-carboxylate (ACC) into ammonia and α-ketobutyrate can enhance bacterial metabolism in response to sustain plant health. The detection of *acd*A gene in the genome of bacteria may perhaps uncover their potential in stimulating plant internal responses against environmental stressors. The positive influence of *B. mojavensis* isolated from pea (*Pisum sativum*) on plant growth due to possession of *acd*S gene has been documented [53]. IAA biosynthesis in a culture medium depends on the utilization of precursory factor (tryptophan) by the endophytic bacteria. Various bacteria isolated from the root endosphere promote plant growth and soil health and the diverse endophytic bacterial community is known to produce IAA and other plant growth-promoting traits. Genome analysis of strain T4S predicts the presence of *dha*S and *trp*BCDES genes that are involved in L-tryptophan production, IAA production, and IPA pathway. The presence of IAA-enzyme coding genes in the genome of endophytic bacteria confirms their involvement in IAA biosynthesis. Hence, identification of plant growth-promoting genes *trp*ABD, *trp*ABCDE, and *trp*BE from *Sphingomonas* sp. LK11, *Pseudomonas aeruginosa* B18, and *Enterobacter roggenkampii* ED5 from the sugarcane root endosphere have been documented [25]. The putative candidate genes and gene families identified in *B. cereus* T4S have the potential for directing further experimental tests of their roles in sunflower growth similar to previous experiments examining cucumber yield in *B. subtilis* MBI 600 [29].

Additionally, studies on the possible novel genes that involve in IAA synthesis through the tryptophan-dependent indole-3pyruvate (*IPy*A) pathway by *B. amyloliquefaciens* SQR9a have been studied [54]. These genes are primarily involved in the conversion of IAA synthesis precursor (tryptophan) *IPy*A. Furthermore, pyruvate decarboxylase, pyruvate oxidase, and phenolic acid decarboxylase participate in the conversion process of *IPy*A to indole-3-acetaldehyde (IAAld). The observed enzyme coding gene (aldehyde dehydrogenase genes, *dha*S) in strain T4S has also been implicated in the conversion process of IAAld to IAA, which further corroborates the results obtained from the genomic analysis of *B. amyloliquefaciens* SQR9a [54].

Some industrially and biotechnologically important enzymes from bacterial endophytes have been reported [55], and their vital role in plant defense mechanisms against phytopathogen resistance is known. Examples of these enzymes include chitinase, cellulase, glucanase, amylase, catalase, and peroxidase. In this study, various genes (*lon*B, *amy*S, and *pul*A) that involved enzyme synthetic pathways were detected in strain T4S, thus suggesting the ability to stimulate plant immune response.

Various genes involved in the protection against oxidative and nitrosative stress were detected in strain T4S. The survival of plants inoculated with bacterial endophytes that thrive under high saline environments could suggest their possible use as bioinoculants to boost salt tolerance in plants [50]. The result agrees with the earlier reports on the osmotic stress tolerance genes in *B. megaterium* STB1 [30]. However, the authors further stressed that the presence of cold and heat shock genes (*csp* and *hsp*), and chaperones in the genome of strain *B. megaterium* STB1 contributed to RNA and DNA stabilization for easy expression (translation and transcription) under cold or heat stress conditions. Although, chaperone encoding genes were not detected in strain T4S.

The participatory genes in the biological control and synthesis of volatile compounds form an essential component of the bacterial cell. The detection of *ilv*ABCDEN gene coding for 2,3-butanediol could be linked to induction of systemic resistance in plants, and protect bacterial cells at low pH and other deleterious metabolites secreted from the root rhizosphere.

Commercialization of known strains of *Bacillus* species of the same ecotype and their activities in sustaining plant growth stand promising as a source of metabolic compounds, such as macrolactin, difficidin, and bacillaene [56]. The genomic information of strain T4S account for most similar known gene clusters specific for secondary metabolite biosynthesis that involves various biological functions in the host plants. Similar metabolic compounds found in the genome of *B. velezensis* have been reported to enhance plant growth and disease suppression [57]. Furthermore, it has been postulated that the genomic similarity of *Bacillus* species could contribute to their interaction mechanisms based on inherent genes involved in biofilm formation [58]. Information regarding the identification of antibiotic compounds in the genome of *Bacillus* and *Streptomyces* has been documented, thus suggesting their exploration as a source of biocontrol agent [59].

Interestingly, the genome of *B. cereus* T4S contains six important genes coding for siderophore, terpene, betalactone, and non-ribosomal peptide (NRPS), such as petrobactin, molybdenum cofactor, fengycin, and bacillibactin, bacitracin, and zwittermicin A, respectively. All these compounds contributed to the biocontrol activity of strain T4S. Siderophore, terpene, betalactone, and NRPS have been implicated in the control of phytopathogens and boosting plant response to environmental stressors [60]. Fengycin production by strain T4S stands as potent antifungal compounds that can cause cell membrane lysis and eventually cell death. Here, the result obtained in this study corroborates the findings of Teixeira et al. [58] who reported NRPS (bacillibactin) from the genome of *B. velezensis* CMRP 4490. Half of the similar known clusters in strain T4S had no similar identity in the database. Hence, the unidentified or yet-to-be described products have created opportunities for future research. Different gene clusters encoding for siderophore, NRPS, and polyketide, also with other novel gene clusters were found in strain T4S. The presence of these metabolite compounds in strain T4S can serve as a source of biocontrol agents for plant protection. However, several findings on the antimicrobial compounds from *B. velezensis* against pathogenic fungi have been documented [58], but less is known on endophytic bacterium *B. cereus* T4S.

In our view, the aforementioned genes involved in various metabolic pathways is the first report on the genome sequencing of sunflower-associated endophytic strain T4S with novel plant growth-promoting attributes and biocontrol potential, which suggest their commercialization prospect in South Africa. A series of several genes identified may play an important role in root colonization to enhance plant growth and immunity. To confirm the implication of gene products from strain T4S on plant growth promotion, further research by chemical analytical methods is required. Hence, optimization of strain T4S based on their gene expression for use under greenhouse experiments may contribute to agricultural productivity. The effect of pure culture of strain T4S upon inoculation revealed better yield in inoculated sunflower plants than un-inoculated. This might be due to the viability and adherence of the bacterial to the surface of the seeds. It is important to state here that the sunflower seed bacterized with strain T4S germinated faster compared to the non-inoculated (Figure 4). Bacterization of sunflower seeds with strain T4S led to faster and higher growth under greenhouse experiments. Enhanced below and aboveground sunflower parameters can be explained by the suggestion that strain T4S which colonized roots interdependently enhanced root development in the uptake of nutrients from the soil for plant nutrition. In addition, the enhanced sunflower yield in terms of nutrient acquisition from soil could be a consequence of strain T4S adherence to the seeds before planting. Our findings are in agreement with the reports of Soni et al. [35] and Prashanth and Mathivanan [61] on cumin and groundnut seeds bacterized with *Paenibacillus polymyxa* HK4 and *B. licheniformis* MML2501, which showed a higher germination rate of bacterized seedlings in comparison to control. Moreover, soil physicochemical parameters and the ability of bacterial endophytes to secrete phytohormones serve as factors contributing to plant development. The CMC used as a binder help to retain water level on the seed surface as well as the source of nutrients, and their extensive use in food and pharmaceutical industries is known [62]. A significant increase in growth parameters of some inoculated plants with *Bacillus* spp. has been reported [63]. Thus, the results obtained from this study corroborate with the findings of Singh et al. [64] who earlier documented on the increase in shoot and root length of sunflower inoculated with *Rhizobium* SF48. Furthermore, improvement in the yield of sunflower treated with plant growth-promoting bacteria, as reported by different researchers, can ensure sustainable sunflower production [1,65]. The increase in the yield of sunflower inoculated with strain T4S can be linked to their genetic composition, functionally in the synthesis of phytohormones which modulate bacteria activities in enhancing sunflower yield. Furthermore, a report by Zahra et al. [66] revealed the effect of sunflower seed inoculation by endophytic actinobacteria and higher sunflower yield (plant length, weight, and flower diameter) than that of non-inoculated sunflower under greenhouse and field experiments. The authors recommended possible exploration of these bacteria in producing biofertilizers for enhanced sunflower growth, which is the focus of this study.

## 4. Materials and Methods

### 4.1. Isolation of Sunflower Root Endosphere Associated Endophytic Bacterium, B. cereus T4S

Sunflower roots were sourced from farmlands in Lichtenburg, South Africa (26°4′31.266″ S, 25°58′44.442″ E) in February 2020. Bacteria isolate was isolated from the sunflower root endosphere on Luria Bertani (LB) agar medium following the method described by Annapurna et al. [45]. Briefly, the plant roots obtained were placed in zip-lock bags on an ice-box for transportation to the laboratory within 4 h of collection for further analysis. For bacterial isolation, the roots were cut into small sizes and surface-sterilized using disinfectants. From the serial dilution process, 0.1 mL from 10^6^ dilutions were aseptically dispensed into sterile Petri dishes and pour plated with sterilized LB medium. Then incubated at 28 °C for 24 h and then checked for colony growth. The pure isolate was obtained by streaking on fresh LB agar plates. For cultural and morphological characterization, various biochemical tests were performed. The isolate was kept on agar slants at 4 °C for further analysis. Furthermore, the isolate was preserved in 30% (*v*/*v*) glycerol at −20 °C.

### 4.2. Morphological and Biochemical Characterization of Bacterial Isolate

The cultural attributes of the bacteria isolate were evaluated on LB media after incubation at 28 °C for 24 h. The cultural morphology of bacterial isolate was visualized under a light microscope (ECLIPSE E200; Nikon, Japan). Biochemical tests, such as sugar fermentation tests (mannitol, glucose, sucrose, maltose, fructose, galactose, and raffinose), catalase test, oxidase test, citrate test, nitrate utilization test, starch, and casein hydrolysis were performed.

### 4.3. DNA Extraction, Polymerase Chain Reaction, and Molecular Identification of the Bacterial Isolate

The genomic content of the pure bacterial isolate was extracted using a commercial Quick-DNA^TM^ Miniprep Kit specific for fungi or bacteria (Zymo Research, Irvine, CA, USA; Cat. No. D6005), following the manufacturer’s guide. The concentration of the extracted DNA (ng/μL) was measured using a NanoDrop Lite spectrophotometer (ThermoFisher Scientific, Carlsbad, CA, USA) and stored at −80 °C. The determination of 16 S rDNA nucleotide sequences of the identified bacterial isolate was achieved using the amplified PCR products. The amplification process was initiated using universal oligonucleotide primers, 27F (5′-AGAGTTTGATCCTGGCTCAG-3′) and 1492R (5′-TACGGTTACCTTGTTACGACTT-3′). A total of 25 µL reaction volume for each bacterial isolate composed of 12.5 μL OneTaq 2× MasterMix with the Standard Buffer, 1 μL for each primer, 2 μL genomic DNA, and 9.5 μL nuclease-free water were used for PCR amplification on DNA Engine DYAD^TM^ Peltier Thermal Cycler (BIO-RAD, C1000 Touch^TM^, Hercules, CA, USA) [67]. The PCR conditions were set to initial denaturation at 94 °C for 5 min and 35 cycles of amplification. Furthermore, the denaturation temperature was set at 94 °C for 30 s, annealing at 50 °C for 30 s, extension at 68 °C for 1 min, and a final extension at 68 °C for 10 min.

After amplification, PCR products were checked in 2% agarose gel prepared in 1 × TAE buffer, and heat in microwave for 4 min. After cooling, 10 μL ethidium bromide was added for the electrophoresis. The band size of the amplicons was determined using a 1 kb molecular marker. After that, the gel was visualized in a Chemidoc^TM^ imaging system (BIO-RAD Laboratories, Hercules, CA, USA). Finally, 20 µL of the PCR product of the bacterial isolate was placed in an ice-box pack and sent for sequencing at Inqaba Biotechnical Industries (Pty) Ltd., Pretoria, South Africa. 16S rDNA sequences for the bacterial isolate were submitted to GenBank on the NCBI online server and were assigned an accession number. The strain code and accession number of the 16S rRNA gene sequence data of the identified bacterial endophytes deposited in the GenBank are T4S and MW265423, respectively.

### 4.4. Whole-Genome Sequencing (WGS)

The extracted genomic DNA of strain T4S was fragmented using an enzymatic approach (NEB Ultra II FS kit, Ipswich, MA, USA). The fragmented DNA was selected according to size range (200–700 bp) using AMPure XP beads. Subsequently, each fragment of DNA was end-repaired and ligated on Illumina-specific adapter sequences. Furthermore, the indexing of each sample and selection based on the size in the second step was performed. The quantity of samples at dilution of standard concentration to 4 nM was determined using a fluorometric method. After that, sequencing was performed using a NextSeq mid-out kit (300 cycles) on Illumina’s NextSeq platform, following a guideline as described by the manufacturer. The resulting 400 Mb of data (2 × 150 bp long paired-end reads) were obtained for each sample.

WGS analysis was performed by submitting each sequence (FASTQ file) to the predictive biology online server and data science platform, KBase (https://kbase.us/) (accessed on the 23 July 2021) [68]. First, sequences were uploaded for read processing, and read quality assessment was achieved using FastQC (version 0.11.5) [69]. The removal of sequence adaptor and low-quality bases of the paired-end Illumina raw sequence reads were performed with trimmomatic (version 0.36) [70] to obtain high-quality sequence reads. Furthermore, sequence reads were assembled with SPAdes (version 3.13.0) [71]. After assembly, contigs annotation was performed using RASTtk (Rapid Annotations using Subsystems Technology toolkit—version 1.073) online server to categorize the distribution and functions of the predicted genes into a subsystem (https://rast.nmpdr.org/) (accessed on the 26 June 2021). The bioinformatics analysis was performed using default settings. The prediction of functional protein-coding genes (PCG) was obtained from the genomic protein output after processing in NCBI. Metabolic pathways of biomolecules were obtained from KEGG (Kyoto Encyclopedia of Genes and Genomes) on RAST (https://rast.nmpdr.org/rast.cgi/) (accessed on the 26 June 2021). The circular genome visualization was obtained from kbase (https://kbase.us/) (accessed on the 23 July 2021), while the phylogeny analysis was performed using MrBayes (http://www.phylogeny.fr/one_task.cgi?task_type=mrbayes) (accessed on the 23 July 2021) (version 3.2.6) [68,72]. Secondary metabolites were determined by antiSMASH (version 6.0.0) (https://antismash.secondarymetabolites.org) (accessed on the 26 June 2021) [73].

### 4.5. Plant Growth-Promoting Screening

#### 4.5.1. Phosphate Solubilization Screening

The qualitative screening of *B. cereus* T4S for the solubilization of phosphate was performed according to the modified method of Khan et al. [74]. For the quantitative assay, phosphate solubilization tendencies of the bacteria isolate were performed by inoculating 10 mL sterile Pikovskaya broth in 50 mL Falcon tubes with 0.1 mL (10^6^ CFU/mL) freshly grown bacterial culture, incubated at 30 °C for 120 h at 180 rpm on a rotary shaker machine. Bacterial supernatant was obtained after cold centrifugation (4 °C) of 10 mL bacterial cultures at 10,000 rpm for 5 min. Four milliliters (4 mL) of the color reagent [(1:1:1:2 ratio 3M H_2_SO_4_, 10% (*w*/*v*) ascorbic acid, 2.5% (*w*/*v*) ammonium molybdate and distilled water], were added to 10% (*w*/*v*) of 5 mL trichloroacetic acid inside test tubes. The tubes were allowed to stand for 15 min upon incubation at room temperature. The quantity of phosphate content was measured according to phosphomolybdate, a blue method at an absorbance of 820 nm. The phosphate solubilization potential by the bacterium in the Pikovskaya broth was determined from the phosphate (KH_2_PO_4_) standard curve. Medium without bacterial inoculation serves as the control.

#### 4.5.2. Siderophore Screening

Siderophore producing ability of *B. cereus* T4S was performed on chrome azurol S (CAS) medium following the methods of Khan et al. [74] with little modifications. The quantity of siderophore produced was determined by inoculating LB broth solution containing CAS with 0.1 mL of 24-h old bacterial culture and incubated at 180 rpm on a rotary shaker for 164 h. Centrifugation of bacterial culture suspension was achieved at 8000× *g* for 10 min. Half a milliliter of the cell filtrate was added to 0.5 mL CAS reagent and properly mixed, then incubated for 120 s at room temperature. The quantity of siderophore released was measured at 630 nm using a spectrophotometer (Thermo Spectronic, Merck Chemicals, Pretoria, South Africa). The siderophore estimate was obtained from the regression equation of the standard curve.

#### 4.5.3. Exopolysaccharide (EPS) Test

*Bacillus cereus* T4S was qualitatively screened for EPS production following the modified method of Igiehon et al. [16]. The square-sized sterile Whatman filter paper No. 1 was aseptically and gently placed onto sterile molten LB agar plates. Two microliters (2 μL) of freshly grown 24-h bacterial culture were directly inoculated on the surface of the filter paper placed inside agar plates supplemented with 10% sucrose with pH adjusted to 7. Subsequently, the inoculated plates were incubated at 28 °C for 5 days. After that, the formation of mucoid colonies around the square-size filter paper depicted EPS production.

#### 4.5.4. Indole Acetic Acid Production

Indole acetic acid synthesis by *B. cereus* T4S was conducted according to Gutierrez et al. [75]. Ten milliliters (10 mL) LB broth supplemented with tryptophan were aseptically inoculated with 100 µL freshly grown bacterial culture (10^6^ CFUml^−1^) and incubated at 28 °C for 7 days at 120 rpm in a rotary shaker incubator (SI-600, LAB Companion, Seoul, Korea). Following incubation, the bacterial suspension was cold centrifuged (4 °C) at 10,000 rpm for 5 min to obtain a cell-free supernatant layer. From the crude extract, 1 mL of supernatant was measured into a clean tube, and 2 mL Salkowski reagent (1:30:50 ratio of 0.5 M FeCl_3_ solution: 95% *w*/*w* sulfuric acid: distilled water) was added. Then, a drop of 10 mM orthophosphoric acid was also added to the mixture and incubated for 10 min at room temperature for color development. The appearance of pink coloration in tubes after incubation in the dark indicated a positive result. An un-inoculated tube serves as the control. From the reacting mixture and control tube after incubation, the absorbance was determined at 530 nm using UV-spectrophotometer (ThermoFisher Scientific, Carlsbad, CA, USA). IAA concentration was evaluated from the IAA gradient standard curve (SC).

### 4.6. Inoculum Preparation and Seed Treatment for the Greenhouse Experiment

#### 4.6.1. Inoculum Preparation and Seed Treatment

A seed inoculation assay was used to facilitate bacterial adherence to the disinfected sunflower seeds. The effectiveness of sunflower seed inoculation was performed following the methods of Ullah et al. [76]. The bacterial inoculum size in LB broth at 24-h incubation was standardized to 0.5 (10^6^ CFU/mL) at OD_600_. Surface sterilization of the seeds was performed by washing in sterile distilled water to remove floating-unhealthy seeds and dirt, and disinfected in 70% ethanol for 3 min, followed by 3% hypochlorite for 1 min, then immersed in 70% alcohol for 2 min with final washing with sterile distilled water. Prepared LB broth inoculated with fresh bacterial culture was incubated on shaker incubator machine at 180 rpm for 24 h. The bacterial cells in the broth culture were harvested by cold centrifugation (4 °C) at 8000× *g* for 10 min to obtain the pelletized cells and then washed in 0.85% normal saline solution. The centrifugation and washing of the pellets were performed under sterile conditions. The sterilized seeds were suspended in a bacterial liquid medium (suspension) containing 1% (*v*/*w*) carboxymethyl cellulose (CMC) in a 250 mL flask and agitated for 60 min. The CMC serves as a sticker that causes the adhesion of bacterial mass to the seeds. The seeds suspended in sterile distilled water without bacterial inoculum serve as control.

#### 4.6.2. Greenhouse Experimental Study

The soil used for the experiment was sourced from agricultural farmlands in North-West University, Mafikeng Campus. Soil debris and other plant materials were removed, air dry, and sieved with a 2 mm micro stainless steel mesh sieve, and the soil was placed inside autoclavable plastic bags and sterilized at 121 °C for 15 min. This step was repeated three times to ensure all spore formers, vegetative cells, and forms of life were eliminated. The level of soil sterility after autoclaving was tested by plating on LB agar. Sterilized soil was allowed to cool for 2 days after which 10 kg of soil was aseptically transferred into plastic pots.

The inoculated and non-inoculated pots were arranged randomly in a complete randomized design (CRD) with 8 replicates for each treatment at a 10 cm distance apart in a greenhouse under natural light. The plastic pots measured 34 cm in diameter and 29 cm tall were washed with sterile water and sterilized with 15% sodium hypochlorite solution before filling with 15 kg dry-sterilized loamy soil. The pots containing the sterile soil were moistened with 500 mL sterilized water before sowing. Ten seeds were sown per pot at a depth of 1.5 cm. After seed emergence, i.e., at 8 days, thinning was performed, leaving one sunflower seedling in a pot. Growing sunflower seedlings were maintained on a day-night cycle of 13–14 h natural light, a temperature of 30 ± 2 °C, and relative humidity of 85%. The pots were moistened with an equal amount of water and maintained daily. Pots containing seedlings without inoculation serve as control. The plants were harvested at maturity after 132 days of planting.

#### 4.6.3. Sunflower Morphological Parameters Below-and-Above-Ground Level

The data collected based on sunflower morphological parameters after harvesting included the number of lateral roots, root number, fresh weight, root weight (dry), shoot weight (dry), taproot length, etc. These parameters were considered following the method described by Igiehon et al. [16]. After harvesting from a greenhouse, sunflower plants were taken to the laboratory. The soil adhering to plant roots was thoroughly washed under running sterile water. The fresh weight of roots and shoots was measured using a weighing balance (Wagi Elektroniczne, Radom, Poland). Furthermore, plant roots and shoots were oven-dry at 60 °C for 24 h and re-weighed on a weighing balance for the determination of roots and shoot dry weights.

#### 4.6.4. Determination of Sunflower Yield Parameters

The sunflower yield parameters were obtained above plant level. The parameters considered include seed weight (fresh and dry), total seed weight (dry), head weight (fresh and dry). After drying, sunflower seeds were manually separated from the sunflower head containing seeds. Seeds were dried in an oven at 60 °C for 24 h, and dry weight was measured on a weighing balance. The plant samples were kept in plastic bags for further analysis. The whole experiment was repeated in triplicate for each treatment.

#### 4.6.5. Soil Variable Analysis

Approximately 0.5 kg of sieved soil was used for soil physical and chemical parameters determination [16]. The chemical parameters such as a percentage (%) silt, clay, and sand, and chemical parameters such as magnesium, iron, potassium, phosphorus, manganese, organic carbon, organic matter, pH, and total nitrogen were evaluated.

### 4.7. Statistical Analysis

The analysis of data from this study was analyzed using SPSS version 16.0. One-way analysis of variance (ANOVA) was performed for the data, followed by Duncan test at 5% level of significance to determine differences between mean. For each treatment, generated data were presented as arithmetic means ± standard deviation.

## 5. Conclusions

From this study, it is evident that *B. cereus* T4 S could be projected into agricultural and crop management based on the possession of putative genes annotated to different biological pathways. The presence of these genes can be overviewed from biotechnological perspectives into main functions such as synthesis of growth hormones, volatile organic compounds, and biocontrol agents, metabolism of organic substrates, and solubilization of essential nutrients. Primarily, the abundance of secondary metabolite gene clusters could provide more insights in the search for a new source of antibiotics. Interestingly, the results from this study have provided new information regarding the genomic competence of *B. cereus* T4S associated with sunflower. Furthermore, the previously demonstrated ability of other *B. cereus* strains with strong affinity in the establishment of plant-bacterial interactions based on notable genes involved in motility, biofilm production, chemotaxis, and attachment to plant root surfaces could make *B. cereus* T4S a suitable candidate in the synthesis of bioinoculants with promising biotechnological application in agriculture for improved sunflower yield.

## Figures and Tables

**Table 1:** Plant growth-promoting features of *B. cereus* T4S.

Test	Plant Growth-Promoting Traits				
	IAA (µg/mL)	Siderophore (%)	Phosphate (µg/mL)	Exopolysaccharide	
Qualitative	+	++	+	+++	
Quantitative	11.29 ± 0.01 ^a^	87.30 ± 0.38 ^c^	30.43 ± 0.18 ^b^	ND	
Test	Enzyme Assay				
	Amylase	Cellulase	Xylanase	Mannanase	Protease
Qualitative	−	++	++	++	+
ZOC (mm)	0 ± 0.00 ^a^	50.00 ± 0.01 ^d^	180.00 ± 0.01 ^e^	35.00 ± 0.01 ^c^	4.00 ± 0.01 ^b^

Key: − = negative reaction, + = positive reaction, ZOC—zone of clearance measurement, ND—not determined. Values are represented as mean ± standard deviation of triplicate readings. The superscript (small letters) within the same row represent a significant difference.

**Table 2:** Genes involved in nitrogen fixation and nitrogen metabolism.

Pathway	Gene	Product	Locus Tag
Nitrogen fixation	*nif*3*-like*	nitrogen fixation protein *Nif*3-like	HWX41_RS02785/HWX41_RS08235/HWX41_RS00530
	*nif*U	nitrogen fixation protein *Nif*U	HWX41_RS22890
	*nif*	Flavodoxin	HWX41_RS17075
Nitrogen metabolism	*glt*P	glutamate/aspartate: proton symporter *Glt*P	HWX41_RS16800
	*glt*X	Glutamate—tRNA ligase	HWX41_RS26220
	*glnR*	transcriptional repressor *Gln*R	HWX41_RS05880
	*gln*A *gln*H	type I glutamate—ammonia ligase/glutamine ABC transporter substrate-binding protein *Gln*H	HWX41_RS05885 HWX41_RS23555
Nitrogen regulation	*nad*R	transcription repressor *Nad*R	HWX41_RS02105
Dissimilatory nitrate reduction	*nir*B *nir*D	nitrite reductase (NADH) large subunit nitrate reductase (NADH) small subunit	HWX41_RS13635 HWX41_RS13640
	*nar*I	respiratory nitrate reductase subunit gamma	HWX41_RS13700
	*Nar*H *nar*J	nitrate reductase subunit beta/nitrate reductase molybdenum cofactor assembly chaperone	HWX41_RS13710 HWX41_RS13705
	*nar*	nitrate reductase subunit alpha	HWX41_RS13715
	*nar*	nitrate reductase	HWX41_RS13945
	*nark*	nitrate transporter	HWX41_RS13665
Ammonia assimilation	*glt*X	glutamate-tRNA ligase	HWX41_RS26220
	*glt*P	glutamate/aspartate: proton symporter *Glt*P	HWX41_RS16800

**Table 3:** Genes Involved in Phosphate Solubilization and Transport.

**Pathway**	Gene	Product	Locus Tag
Degradation of phosphonates	*phn*C *phn*F	phosphonate transport system ATP-binding phosphonate metabolism transcriptional regulator *phn*F phosphonate transport system permease protein	HWX41_RS05935 HWX41_RS04040
	*phn*E	phosphonate transport system permease protein	HWX41_RS05940/HWX41_RS05945
	*isp*H	4-hydroxy-3-methylbut-2-enyl diphosphate reductase	HWX41_RS02790
Phosphate transport	*phn*	phosphate/phosphite/phosphonate ABC transporter substrate-binding protein	HWX41_RS05930
	*pst*C *pst*S *pst*S	phosphate transport system permease phosphate transport system substrate-binding protein phosphate transport system substrate-binding protein	HWX41_RS23195 HWX41_RS02875/HWX41_RS23200HWX41_RS02870
	*pst*B	phosphate transport system ATP-binding protein	HWX41_RS02885
	*pst*A	phosphate transport system permease protein	HWX41_RS23190/HWX41_RS02880
	*pho*U	phosphate signaling complex protein *Pho*U	HWX41_RS02890
	*glt*P	glycerol-3-phosphate transporter	HWX41_RS23465
	*ugpC*	sn-glycerol-3-phosphate ABC transporter ATP-binding protein *Ugp*C	HWX41_RS04235/HWX41_RS23895
	*pho*H	phosphate starvation-inducible protein *Pho*H and related proteins	HWX41_RS02710

**Table 4:** Genes involved in iron transport and siderophore production.

Pathway	Gene	Product	Locus Tag
Iron(III) transport	*fbp*A	fur-regulated basic protein *Fbp*A	HWX41_RS15335/HWX41_RS19065
Iron(II) transport	*fet*B	ferrous iron transport protein A	HWX41_RS00335
	*fet*B	iron export ABC transporter permease subunit *Fet*B	HWX41_RS22925
	*feo*B	ferrous iron transport protein B	HWX41_RS00340
Siderophore transport	*fet*B	siderophore ABC transporter substrate-binding protein	HWX41_RS24585

**Table 5:** Genes involved in the modulation of plant hormones.

Pathway	Gene	Product	Locus Tag
ACC catabolism	*acd*A	acyl-CoA dehydrogenase *Acd*A	HWX41_RS21030
Potassium transport	*kdp*A	potassium-transporting ATPase subunit A	HWX41_RS15255
L-tryptophan production; IAA production	*ND*	tryptophan synthase subunit alpha	HWX41_RS17780
	*trp*B	tryptophan synthase subunit beta	HWX41_RS17785
	*trpC*	indole-3-glycerol phosphate synthase *Trp*C	HWX41_RS17795
	*trp*D	anthranilate phosphoribosyltransferase	HWX41_RS17800
	*trp*E	anthranilate synthase component I	HWX41_RS17810
	*trp*S	Tryptophan—tRNA ligase	HWX41_RS18055
IAA production, IPA pathway	*dha*S	aldehyde dehydrogenase *Dha*S	HWX41_RS06485
	*dha*	aldehyde dehydrogenase	HWX41_RS25615
	*dha*	acetaldehyde dehydrogenase (acetylating)	HWX41_RS13995
	*dha*	aldehyde dehydrogenase family protein	HWX41_RS19325
	*dha*	aldehyde dehydrogenase family protein	HWX41_RS06850
	*dha*	aldehyde dehydrogenase family protein	HWX41_RS10480
	*dha*	aldehyde dehydrogenase family protein	HWX41_RS13000
	*dha*	aldehyde dehydrogenase family protein	HWX41_RS17225
	*dha*	aldehyde dehydrogenase family protein	HWX41_RS17590
	*dha*	aldehyde dehydrogenase family protein	HWX41_RS14005
CK biosynthesis and transformation	*mia*A	tRNA (adenosine(37)-N6)-dimethylallyltransferase *Mia*A	HWX41_RS05835
Ammonia production	*nad*E	ammonia-dependent NAD(+) synthetase	HWX41_RS14365

Key: ND—not determined.

**Table 6:** Estimate of secondary metabolite genes in genome of *B. cereus* T4S.

Node Rg	From	To	MSKC		Type	Similarity
Rg 1.1	28,953	42,560	Petrobactin	Other	Siderophore	100%
Rg 3.1	93,263	140,279			NRPS	
Rg 3.2	155,248	165,496			RiPP-like	
Rg 21.1	20,448	75,995			NRPS	
Rg 28.1	22,582	65,892	Bacillibactin	NRP	NRPS	46%
Rg 35.1	1	57,852	Bacitracin	NRP	NRPS	100%
Rg 42.1	16,640	38,493	Molybdenum cofactor	Other	Terpene	17%
Rg 46.1	1	45,939	Zwittermicin A	NRP + Polyketide	NRPS, T1PKS	77%
Rg 62.1	5236	34,100			NRPS-like	
Rg 71.1	8999	19,310			RiPP-like	
Rg 86.1	1	23,433			NRPS	
Rg 87.1	1	17,771			LAP, RiPP-like	
Rg 89.1	1	22,451	Zwittermicin A	NRP + Polyketide	NRPS, T1PKS	7%
Rg 91.1	7584	22,279	Fengycin	NRP	Betalactone	20%

Key: MSKC—Most similar known cluster, Rg—region, NRPS—nonribosomal peptides, NRP—nonribosomal peptide, T1PKS—type 1 polyketide synthases, LAP—lantipeptide, RiPP—ribosomally synthesized and post-translationally modified peptide.

**Table 7:** Sunflower Yield Parameters.

Level	Growth Parameter	Non-Inoculated	Inoculated with *B. cereus* T4S
Belowground	Tap root length (cm)	146.67 ± 58.33 ^a^	158.33 ± 40.20 ^b^
	Tap root width (cm)	5.33 ± 0.58 ^a^	7.00 ± 1.00 ^b^
	Root length (cm)	217 ± 81.22 ^a^	339.67 ± 82.78 ^b^
	Lateral root number	25.67 ± 1.53 ^a^	29.67 ± 1.53 ^b^
	Root wet weight (g)	44.09 ± 16.12 ^a^	65.54 ± 18.41 ^b^
	Root dry weight (g)	8.41 ± 1.55 ^a^	11.43 ± 5.44 ^b^
	Number of roots	935 ± 11.30 ^a^	1209.67 ± 348.26 ^b^
Aboveground	Seed wet weight (g)	0.05 ± 0.01 ^a^	0.25 ± 0.33 ^b^
	Seed dry weight (g)	0.03 ± 0.01^a^	0.035 ± 0.00 ^a^
	Head fresh weight (g)	153.76 ± 13.94 ^a^	165.50 ± 7.55 ^b^
	Head dry weight (g)	41.55 ± 4.27 ^a^	45.92 ± 13.33 ^b^
	Plant wet weight dry (g)	331.04 ± 20.16 ^a^	392.69 ± 8.70 ^b^
	Shoot wet weight (g)	165.82 ± 5.16 ^a^	183.55 ± 13.75 ^b^
	Shoot dry weight (g)	57.28 ± 7.04 ^a^	68.24 ± 22.13 ^b^

Values are represented as means ± standard deviation of triplicate readings. The superscript (small letters) within the same column represent a significant difference.

## Data Availability

From the NCBI database output, the Bioproject number is https://www.ncbi.nlm.nih.gov/bioproject/PRJNA706601, and Sequence Read Archive (SRA) number is https://www.ncbi.nlm.nih.gov/sequencereadarchive/SRR13887969, and BioSample number is https://www.ncbi.nlm.nih.gov/biosample/SAMN18138757. The genome accession number JAFNAY000000000 was assigned to the strain T4S.

## Supplementary Materials

The following are available online at https://www.mdpi.com/article/10.3390/plants10091776/s1, Table S1: Biochemical, cultural characterization of B. cereus T4S: Table S2: Protein secretion systems genes; Table S3: Genes involved in sulfur metabolism; Table S4: Genes involved in motility, biofilm formation, and chemotaxis; Table S5: Genes involved in attachment to plant surfaces; Table S6: Genes involved in the protection against oxidative and nitrosative stress; Table S7: Genes involved in carbohydrate metabolism; Table S8: Genes involved in carbohydrate transport; Table S9: Genes involved in the metabolism of organic acids; Table S10: Genes involved in amino acid metabolism and transport; Table S11: Genes involved in opine and GABA transport and metabolism; Table S12: Genes involved in lignin degradation and degradation against toxic peroxides; Table S13: Genes involved in biological control; Table S14: Physical and chemical analysis of the soil samples.
