# Supplementary material for: Genomic Analysis of Endophytic Bacillus cereus T4S and Its Plant Growth-Promoting Traits
Source: Plants (Basel). 2021 Aug 26;10(9):1776. doi: 10.3390/plants10091776 (PMC8467928; doi:10.3390/plants10091776)
Supplement: Supplementary file 1 [file plants-10-01776-s001.zip › plants-1312873-supplementary.pdf]

Supplementary materials

Genomic Analysis of Endophytic *Bacillus cereus* T4S and Its Plant Growth-promoting Traits

Bartholomew Saanu Adeleke [1], Ayansina Segun Ayangbenro [1] and Olubukola Oluranti Babalola [1*]

[1] Food Security and Safety Niche Area, Faculty of Natural and Agricultural Sciences, North-West University, Private Bag X2046, Mmabatho 2735, South Africa
* Correspondence: olubukola.babalola@nwu.ac.za; Tel.: +27-(0)183892568

Table S1. Biochemical, cultural characterization of *B. cereus* T4S.

Characteristics	Result
Gram reaction	+
Shape	rod
Catalase	+
Starch hydrolysis	+
Nitrate	+
Oxidase	+
Citrate	+
Casein hydrolysis	+
Salt tolerance (% *v/v*) (max)	5.5
Temperature range (°C)	25–45
pH	4–10
Fructose	+
Galactose	+
Sucrose	+
Glucose	+
Xylose	+
Raffinose	+
Mannitol	+
Maltose	+

Key: + = positive reaction.

Table S2. Genes involved in protein secretion systems.

Pathway	Gene	Product	Locus Tag
Sec (secretion) system	*sec*A	preprotein translocase subunit *Sec*A	HWX41_RS21805
	*sec*E/G/Y	preprotein translocase subunit *Sec*E/G/Y	HWX41_RS26260
	*sec*E/G/Y	preprotein translocase subunit *Sec*E/G/Y	HWX41_RS22075
	*sec*E/G/Y	preprotein translocase subunit *Sec*E/G/Y	HWX41_RS26435/HWX41_RS11560
Twin-arginine translocation (Tat) system	*yaj*C	preprotein translocase subunit *Yaj*C	HWX41_RS02190
	*tat*E/*tat*A	protein translocase protein *Tat*E/*Tat*A	HWX41_RS13235
	*tat*C	protein translocase protein *Tat*C	HWX41_RS13230
	*tat*C	twin-arginine translocase subunit *Tat*C	HWX41_RS13230

Table S3. Genes involved in sulfur metabolism.

Pathway	Gene	Product	Locus Tag
Sulfate transport	*cys*C	adenylyl-sulfate kinase	HWX41_RS16845
	sat	sulfate adenylyltransferase	HWX41_RS16850
	*cys*T	sulfate transport system permease protein	HWX41_RS18405
	cys	sulfate transport system substrate-binding protein	HWX41_RS18410/HWX41_RS21870
	*cys*W	sulfate ABC transporter permease subunit *Cys*W	HWX41_RS18400
	*cys*K	cysteine synthase A	HWX41_RS15030/HWX41_RS26760
	*cys*S	cysteine--tRNA ligase	HWX41_RS26230
	*sul*P	sulfate permease	HWX41_RS25435

Table S4. Genes involved in motility, biofilm formation, and chemotaxis.

Pathway	Gene	Product	Locus tag
Chemotaxis	*che*V	chemotaxis protein *Che*V	HWX41_RS15615
	che	chemotaxis protein	HWX41_RS23355
	*che*W	purine-binding chemotaxis protein *Che*W	HWX41_RS15745
	che	methyl-accepting chemotaxis protein	HWX41_RS07060/HWX41_RS22445
Flagella biosynthesis	*flg*D	flagellar basal body rod modification protein *Flg*D	HWX41_RS15635
	*fli*K	flagellar hook-length control protein *Fli*K	HWX41_RS15640
	*flh*B	flagellar type III secretion system protein *Flh*B	HWX41_RS15485
	*flg*B	flagellar basal body rod protein *Flg*B	HWX41_RS15680
	*flh*A	flagellar biosynthesis protein *Flh*A	HWX41_RS15480
	*flg*E	flagellar hook protein *Flg*E	HWX41_RS15630
	*fli*L	flagellar protein export ATPase *Fli*I	HWX41_RS15650
	*flg*G	flagellar basal-body rod protein	HWX41_RS15470
	flg	flagellar motor switch protein	HWX41_RS15660
	*flg*G	flagellar biosynthesis protein *Flg*G	HWX41_RS15685
	*flh*F	flagellar biosynthesis protein *Flh*F	HWX41_RS15475
	*fli*R	flagellar type III secretion system protein *Fli*R	HWX41_RS15490
	*fli*F	flagellar M-ring protein *Fli*F	HWX41_RS15665
	*fli*H	flagellar assembly protein H	HWX41_RS15655
	*fli*Q	flagellar biosynthetic protein *Fli*Q	HWX41_RS15495
	*fli*S	flagellar protein *Fli*S	HWX41_RS15690
	flip	flagellar type III secretion system pore protein *Fli*P	HWX41_RS15500
	*fli*N	flagellar motor switch protein *Fli*N	HWX41_RS15510/HWX41_RS15520
	*fli*C	flagellar basal body rod protein *Flg*C	HWX41_RS15675/HWX41_RS16785
	*fli*Y	flagellar motor switch phosphatase *Fli*Y	HWX41_RS15740
	*fli*E	flagellar hook-basal body complex protein *Fli*E	HWX41_RS15670

	*mot*A	flagellar motor stator protein *Mot*A/Chemotaxis protein *Mot*A	HWX41_RS01775
	*flg*K	flagellar hook-associated protein *Flg*K	HWX41_RS15705
	*fli*M	flagellar motor switch protein *Fli*M	HWX41_RS15515
	*mot*B	flagellar motor protein *Mot*B/Chemotaxis protein *Mot*B	HWX41_RS01780/HWX41_RS04225
	ND	flagellar hook-associated protein 2 / protein 3	HWX41_RS15695 HWX41_RS15700
	ND	flagellin	HWX41_RS15530
	ND	flagellin	HWX41_RS15535
Pilus and fimbriae biosynthesis	ND	isopeptide-forming domain-containing fimbrial protein	HWX41_RS27290
Biofilm formation	*efp*	elongation factor P	HWX41_RS03245
	hfq	RNA chaperone *Hfq*	HWX41_RS05840/HWX41_RS15770
	crp	*Crp*/*Fnr* family transcriptional regulator	HWX41_RS16660

Key: ND—not determined.

Table S5. Genes involved in attachment to plant surfaces.

Pathway	Gene	Product	Locus tag
LPS, Lipid A biosynthesis and modifications	*mur*F	UDP-N-acetylmuramoyl-tripeptide--D-alanyl-D-alanine ligase	HWX41_RS25735
	*mur*A	UDP-N-acetylglucosamine 1-carboxyvinyltransferase	HWX41_RS21065/HWX41_RS21305
	*mur*B	UDP-N-acetylmuramate dehydrogenase	HWX41_RS04775
	*mur*C	UDP-N-acetylmuramate--L-alanine ligase	HWX41_RS00935
	*mur*E	UDP-N-acetylmuramoyl-L-alanyl-D-glutamate--2,6-diaminopimelate ligase	HWX41_RS04750
	*mur*D	UDP-N-acetylmuramoyl-L-alanine--D-glutamate ligase	HWX41_RS04760
	*mur*G	undecaprenyldiphospho-muramoylpentapeptide beta-N-acetylglucosaminyltransferase	HWX41_RS04770
	*gal*E	UDP-glucose 4-epimerase *Gal*E	HWX41_RS21410
	*wec*B	UDP-N-acetylglucosamine 2-epimerase (non-hydrolyzing)	HWX41_RS21750
	ND	UDP-N-acetylmuramate dehydrogenase	HWX41_RS22310
	ND	UDP-glucosyltransferase	HWX41_RS11130
	ND	UDP-galactose-lipid carrier transferase	HWX41_RS15090
	ND	UDP-glucose/GDP-mannose dehydrogenase family protein	HWX41_RS25510
	*glm*U	bifunctional UDP-N-acetylglucosamine diphosphorylase	HWX41_RS26655
	*glm*U	glucosamine-1-phosphate N-acetyltransferase *Glm*U	HWX41_RS26655
	*upp*P	bacitracin resistance undecaprenyl-diphosphatase, undecaprenyl-diphosphate phosphatase *Upp*P	HWX41_RS17035 HWX41_RS27450
LPS, O-antigen biosynthesis	*rfb*D	dTDP-4-dehydrorhamnose reductase	HWX41_RS17900
	*rfb*B	dTDP-glucose 4,6-dehydratase	HWX41_RS17905
	*rfb*C	dTDP-4-dehydrorhamnose 3,5-epimerase	HWX41_RS17910

Key: ND—not determined.

Table S6. Genes involved in the protection against oxidative and nitrosative stress.

Pathway	Gene	Product	Locus tag
Degradation of superoxide anion radicals	*sod*A	superoxide dismutase	HWX41_RS20530/HWX41_RS02855
	*sod*A	superoxide dismutase family protein	HWX41_RS23120
	*sod*A	superoxide dismutase	HWX41_RS16575
Degradation of hydrogen peroxide	*kat*B	catalase	HWX41_RS18175
	ND	manganese catalase family protein	HWX41_RS19840
	kat	catalase	HWX41_RS07625/HWX41_RS09520
Degradation of hydrogen peroxide and organic hydroperoxides	*tpx*	Thiol peroxidase	HWX41_RS01185
	bcp	thioredoxin-dependent thiol peroxidase	HWX41_RS25065
	*osm*C	*Osm*C family protein (peroxiredoxin osmc)	HWX41_RS23535
	*ahp*C	Peroxiredoxin	HWX41_RS24440
	*ahp*F	Alkyl hydroperoxide reductase subunit F	HWX41_RS24435
	ND	alkylphosphonate utilization protein	HWX41_RS25420
	ND	organic hydroperoxide resistance protein	HWX41_RS01930
Detoxification of peroxidized compounds	*bsa*A	glutathione peroxidase	HWX41_RS13745
	grx	glutaredoxin family protein	HWX41_RS22030/HWX41_RS04375
	grx	glutaredoxin family protein	HWX41_RS19015
NOS resistance	ND	nitric oxide synthase oxygenase	HWX41_RS20535
Hydroxylamine reduction	*hcp*	hydroxylamine reductase	HWX41_RS06975
ROS resistance	ND	PQQ-dependent sugar dehydrogenase	HWX41_RS11455

Key: ND—not determined.

Table S7. Genes involved in carbohydrate metabolism.

Pathway	Gene	Product	Locus tag
Pentose phosphate	*tkt*	transketolase	HWX41_RS05950
EMP	*glc*K	glucokinase	HWX41_RS02915
Glycolysis	*glc*U	glucose uptake protein *Glc*U	HWX41_RS00785
	ND	glucose-6-phosphate isomerase	HWX41_RS23165
	ND	glucose 1-dehydrogenase	HWX41_RS19280/HWX41_RS00790
	ND	glucose-6-phosphate dehydrogenase	HWX41_RS08270/HWX41_RS27525
	*pfk*A	6-phosphofructokinase	HWX41_RS01390
	*pfk*B	1-phosphofructokinase	HWX41_RS05815
	ND	fructose-bisphosphate aldolase	HWX41_RS21060
	ND	glyceraldehyde-3-phosphate dehydrogenase	HWX41_RS01470
	gap	type I glyceraldehyde-3-phosphate dehydrogenase	HWX41_RS22040
	*gap*N	NADP-dependent glyceraldehyde-3-phosphate dehydrogenase	HWX41_RS19640
	ND	phosphoglycerate kinase	HWX41_RS22045
	*gpm*L	2,3-bisphosphoglycerate-dependent phosphoglycerate mutase	HWX41_RS22055
	*mtn*W	2,3-diketo-5-methylthiopentyl-1-phosphate enolase	HWX41_RS04125
	*mtn*A	S-methyl-5-thioribose-1-phosphate isomerase	HWX41_RS04145
	*pdh*A	pyruvate dehydrogenase E1 component subunit alpha	HWX41_RS04455
	*pdh*B	pyruvate dehydrogenase complex E1 component subunit beta	HWX41_RS04460
	*pdh*C	pyruvate dehydrogenase complex dihydrolipoyllysine-residue acetyltransferase	HWX41_RS04465
	pyc	pyruvate carboxylase	HWX41_RS04585
	ND	pyruvate kinase	HWX41_RS08535
Maltose transport	*mal*C	maltosaccharide ABC transporter permease *Mal*C	HWX41_RS04255
	*mal*D	maltosaccharide ABC transporter permease *Mal*D	HWX41_RS04260
	*mal*R	maltose operon transcriptional repressor *Mal*R	HWX41_RS04265
Glucose and glucose-1-phosphate degradation	*gnt*K	gluconokinase	HWX41_RS08285

	*glg*C	glucose-1-phosphate adenylyltransferase	HWX41_RS00075
	*glg*B	1,4-alpha-glucan branching protein *Glg*B	HWX41_RS00070
	*glg*D	glucose-1-phosphate adenylyltransferase subunit *Glg*D	HWX41_RS00080
	*glg*A	glycogen synthase *Glg*A	HWX41_RS00085
	*glg*P	glycogen phosphorylase	HWX41_RS00090
	*glc*U	glucose uptake protein *Glc*U	HWX41_RS00785
	*gal*U	UTP--glucose-1-phosphate uridylyltransferase	HWX41_RS23065/HWX41_RS25505
Glucose transport	*pst*G	PTS glucose transporter subunit IIABC	HWX41_RS04055
	*pst*H	phosphocarrier protein HPr	HWX41_RS04060
Fructose degradation	*pfk*A	6-phosphofructokinase	HWX41_RS01390
	*pfk*B	1-phosphofructokinase	HWX41_RS05815
	fsa	fructose-6-phosphate aldolase	HWX41_RS23420
Ribose degradation	*rbs*K	ribokinase	HWX41_RS23445
	*doe*C	deoxyribose-phosphate aldolase	HWX41_RS14830
	*rpi*A	ribose 5-phosphate isomerase A	HWX41_RS10690
	*rpi*B	ribose 5-phosphate isomerase B	HWX41_RS21150
	*rbs*D	D-ribose pyranase	HWX41_RS23440
Ribose transport	*rbs*B	ribose ABC transporter substrate-binding protein *Rbs*B	HWX41_RS23425
	*rbs*C	ribose ABC transporter permease	HWX41_RS23430
	*rbs*A	ribose ABC transporter ATP-binding protein *Rbs*A	HWX41_RS23435
	*rbs*R	ribose operon transcriptional repressor *Rbs*R	HWX41_RS23450
Hexose metabolism	*ND*	aldose 1-epimerase family protein	HWX41_RS15905
GDP-mannose degradation	*ppa*X	GDP-mannose pyrophosphatase *Ppa*X	HWX41_RS21930
	*deo*B	phosphopentomutase	HWX41_RS03865
Trehalose degradation	*tre*C	alpha, alpha-trehalase	HWX41_RS23595
	treC	alpha,alpha-trehalase	HWX41_RS0424
D-galactosamine and N-acetyl-D-galactosamine degradation	*nag*A	N-acetylglucosamine-6-phosphate deacetylase	HWX41_RS04030
	*nag*A	N-acetylglucosamine-6-phosphate deacetylase	HWX41_RS04030
	*nag*B	glucosamine-6-phosphate deaminase	HWX41_RS04035
	*pfk*A	6-phosphofructokinase	HWX41_RS01390
Myo-inositol degradation	*ND*	inositol-1-monophosphatase protein family	HWX41_RS04530
	*iol*E	myo-inosose-2 dehydratase	HWX41_RS07140
	*iol*G	inositol 2-dehydrogenase	HWX41_RS07120

	*iol*D	3D-(3,5/4)-trihydroxycyclohexane-1,2-dione acylhydrolase (decyclizing)	HWX41_RS07135
	*iol*C	5-dehydro-2-deoxygluconokinase	HWX41_RS07125
	ND	5-deoxy-glucuronate isomerase	HWX41_RS07155
	ND	methylmalonate-semialdehyde dehydrogenase (CoA acylating)	HWX41_RS12755
D-gluconate degradation	*gnt*K	gluconokinase	HWX41_RS19365/HWX41_RS08285
D-gluconate transformation to 2-keto D-gluconate (2-KG)	*gnt*P	gluconate permease *Gnt*P	HWX41_RS19360
	*gnt*R	gluconate operon transcriptional repressor *Gnt*R	HWX41_RS19370
2KG to 6-phospho-D-gluconate	*dhb*A	2,3-dihydro-2,3-dihydroxybenzoate dehydrogenase	HWX41_RS12675
	*dhb*C	isochorismate synthase *Dhb*C	HWX41_RS12670
L-lactate degradation	*ND*	L-lactate dehydrogenase	HWX41_RS22670
	ND	L-lactate dehydrogenase	HWX41_RS00065
	ND	L-lactate dehydrogenase	HWX41_RS14695
L-lyxose, 2,3-diketo-L-gulonate, L-ascorbate degradation	*ND*	L-ribulose-5-phosphate 4-epimerase	HWX41_RS05030
D-tagatose degradation	*nag*A	N-acetylglucosamine-6-phosphate deacetylase	HWX41_RS04030
Degradation of Beta-Glucosides (e.g. cellobiose, salicin, arbutin, esculin)	*cel*F	6-phospho-beta-glucosidase	HWX41_RS21710
Cellobiose transport	*cel*B	PTS cellobiose transporter subunit IIC	HWX41_RS21665/HWX41_RS21700
	*cel*F	6-phospho-beta-glucosidase	HWX41_RS21710
Degradation of unknown glucosides	*ND*	glycosyl hydrolase	HWX41_RS15345/HWX41_RS20465
	ND	glycoside hydrolase/lysozyme	HWX41_RS05625
	ND	glycoside hydrolase/lysozyme	HWX41_RS06965
	ND	glycoside hydrolase/lysozyme	HWX41_RS09710
	ND	glycoside hydrolase/lysozyme	HWX41_RS14465

Chitin degradation	***ND***	chitobiase/beta-hexosaminidase C-terminal domain-containing protein	HWX41_RS18500

Key: ND—not determined.

Table S8. Genes involved in carbohydrate transport.

Pathway	Gene	Product	Locus tag
Maltose/maltodextrin and multiple sugars transport	*mal*R	maltose operon transcriptional repressor *Mal*R	HWX41_RS04265
Trehalose-specific EIIBC component	*tre*P [1]	PTS system, trehalose-specific EIIBC component	HWX41_RS23600
	*tre*C	alpha,alpha-phosphotrehalase	HWX41_RS23595
	*tre*R	trehalose operon repressor	HWX41_RS23605
	bcr	*Bcr/Cfl*A family efflux MFS transporter	HWX41_RS26020
	ND	DHA2 family efflux MFS transporterpermease subunit	HWX41_RS01545/HWX41_RS04310<br>HWX41_RS09945/ HWX41_RS12645<br>HWX41_RS12645/HWX41_RS15080
	ND	DHA2 family efflux MFS transporter permease subunit	
	ND	DHA2 family efflux MFS transporter permease subunit	
	ND	peptide MFS transporter	HWX41_RS23330
	ND	OFA family MFS transporter	HWX41_RS12690
Sugar acids transport	*lld*P	L-lactate permease	HWX41_RS21585/HWX41_RS17765

Key: ND—not determined.

Table S9. Genes involved in the metabolism of organic acids

Pathway	Gene	Product	Locus Tag
TCA cycle	*ace*A	isocitrate lyase	HWX41_RS18305
	*prp*B	methylisocitrate lyase	HWX41_RS12770
	*ace*B	malate synthase A	HWX41_RS18310
	icd	NADP-dependent isocitrate dehydrogenase	HWX41_RS01420
	*odh*A	2-oxoglutarate dehydrogenase E1 component	HWX41_RS17695
	*odh*B	2-oxoglutarate dehydrogenase complex dihydrolipoyllysine-residue succinyltransferase	HWX41_RS17700
	*gab*T	4-aminobutyrate--2-oxoglutarate transaminase	HWX41_RS19330/HWX41_RS24350
		adenylosuccinate synthase	HWX41_RS20390
	*pur*B	adenylosuccinate lyase	HWX41_RS24180
	*arg*G	argininosuccinate synthase	HWX41_RS01225/HWX41_RS06510
	*arg*H	argininosuccinate lyase	HWX41_RS01230
	*sdh*C	succinate dehydrogenase cytochrome B558	HWX41_RS01750
	*sdh*A	succinate dehydrogenase flavoprotein subunit	HWX41_RS01755
	ND	succinate CoA transferase	HWX41_RS07255
	*sdh*B	succinate dehydrogenase iron-sulfur subunit	HWX41_RS01760
	*fum*A	fumarate hydratase class I	HWX41_RS24845
	*fum*C	fumarate hydratase class II	HWX41_RS15315
	mdh	malate dehydrogenase	HWX41_RS01425
	*cit*Z	citrate synthase	HWX41_RS01415
	*mmg*D	citrate synthase	HWX41_RS12780
	mend	2-succinyl-5-enolpyruvyl-6-hydroxy-3-cyclohexene-carboxylic-acid synthase	HWX41_RS00135
	*men*H	2-succinyl-6-hydroxy-2,4-cyclohexadiene-1-carboxylate synthase	HWX41_RS00140
	*men*B	1,4-dihydroxy-2-naphthoyl-CoA synthase	HWX41_RS00145
	*men*C	o-succinylbenzoate synthase	HWX41_RS00155
Glyoxylate bypass TCA	*acn*A	aconitate hydratase	HWX41_RS06265
	*ace*A	isocitrate lyase	HWX41_RS18305
	*ace*B	malate synthase	HWX41_RS18310
Formate degradation	*fdh*D	formate dehydrogenase accessory sulfurtransferase *Fdh*D	HWX41_RS15580/HWX41_RS23795
	*fdh*F	formate dehydrogenase subunit alpha	HWX41_RS15595/HWX41_RS23790

Key: ND—not determined.

Table S10. Genes involved in amino acid metabolism and transport

Pathway	Gene	Product	Locus Tag
Methionine degradation	*met*K	methionine adenosyltransferase	HWX41_RS00560
D-alanine degradation	*alr*	alanine racemase	HWX41_RS13960/HWX41_RS25705
	ald	alanine dehydrogenase	HWX41_RS07920
	ald	alanine dehydrogenase	HWX41_RS01270
	ald	alanine dehydrogenase	HWX41_RS13960
	ald	alanine dehydrogenase	HWX41_RS23775
	ald	alanine dehydrogenase	HWX41_RS25230
L-arginine degradation	*spe*A	arginine decarboxylase	HWX41_RS04510
	*lys*A	diaminopimelate decarboxylase	HWX41_RS16865
L-aspartate degradation/conversion	*asp*B	aspartate aminotransferase *Asp*B	HWX41_RS16185
	*asp*A	Aspartate ammonia-lyase	HWX41_RS15170/HWX41_RS23690
	*pan*D	Aspartate 1-decarboxylase	HWX41_RS16205
	*nad*B	L-aspartate oxidase	HWX41_RS02115
	*pry*B	aspartate carbamoyltransferase	HWX41_RS04875
	*dap*G	Aspartate kinase	HWX41_RS05435/HWX41_RS15135
	*asp*S	aspartate--tRNA(Asn) ligase	HWX41_RS13465
L-cysteine degradation	*cys*K	cysteine synthase A	HWX41_RS26760/HWX41_RS15035
	ND	cysteine hydrolase	HWX41_RS26930
	ND	cysteine hydrolase	HWX41_RS02940
	ND	cysteine hydrolase	HWX41_RS09875
Cysteine transformation	*suf*S	cysteine desulfurase	HWX41_RS01140/HWX41_RS22740
	*isc*S	*Isc*S subfamily cysteine desulfurase	HWX41_RS02110/HWX41_RS02290
D-cysteine degradation	*ND*	D-cysteine desulfhydrase	HWX41_RS08870
L-glutamate degradation	*gud*B	NAD-specific glutamate dehydrogenase	HWX41_RS16465
	*pru*A	L-glutamate gamma-semialdehyde dehydrogenase	HWX41_RS24270
L-glutamine degradation	*ND*	glutamate synthase	HWX41_RS25035

	*rac*E	glutamate racemase	HWX41_RS01860/HWX41_RS19645
L-histidine degradation	*hut*H	histidine ammonia-lyase	HWX41_RS06090
	*hut*U	urocanate hydratase	HWX41_RS06095
	*hut*I	Imidazolonepropionase	HWX41_RS06100
	*hut*G	Formiminoglutamase	HWX41_RS06105
	*hut*P	hut operon transcriptional regulator *Hut*P	HWX41_RS06085
	*res*E	sensor histidine kinase *Res*E	HWX41_RS16530
	*res*C	cytochrome c biogenesis protein *Res*C	HWX41_RS16540
	*res*B	cytochrome c biogenesis protein *Res*B	HWX41_RS16545
	*res*A	thiol-disulfide oxidoreductase *Res*A	HWX41_RS16550
	*dcu*S	two-component system sensor histidine kinase *Dcu*S	HWX41_RS23850
L-proline degradation	*ND*	proline dehydrogenase	HWX41_RS22610
	*pro*S	proline--tRNA ligase	HWX41_RS24700
L-tyrosine degradation	*hpp*D	4-hydroxyphenylpyruvate dioxygenase	HWX41_RS25765
	*xer*S	tyrosine recombinase *Xer*S	HWX41_RS25285
Glycine cleavage	*thi*O	glycine oxidase *Thi*O	HWX41_RS20150
	*gcv*H	glycine cleavage system protein *Gcv*H	HWX41_RS22695
	*gcv*T	glycine cleavage system aminomethyltransferase GcvT	HWX41_RS03090
	*gcv*PA	aminomethyl-transferring glycine dehydrogenase subunit 1	HWX41_RS03095
	*gcv*PB	aminomethyl-transferring glycine dehydrogenase subunit 2	HWX41_RS03100
Branched-chain amino acid transport	*asn*	asparagine synthase (glutamine-hydrolyzing)	HWX41_RS13200
	*brn*Q	branched-chain amino acid transport system II carrier protein	HWX41_RS01645
	*brn*Q	branched-chain amino acid transport system II carrier protein	HWX41_RS14100
	*brn*Q	branched-chain amino acid transport system II carrier protein	HWX41_RS14750
	*brn*Q	branched-chain amino acid transport system II carrier protein	HWX41_RS16755
	*brn*Q	branched-chain amino acid transport system II carrier protein	HWX41_RS19845
	*brn*Q		HWX41_RS23325
	*brn*Q		HWX41_RS14100

		branched-chain amino acid transport system II carrier protein	
		branched-chain amino acid transport system II carrier protein	
	dat	D-amino-acid transaminase	HWX41_RS13175

Key: ND—not determine.

Table S11. Genes involved in opine and GABA transport and metabolism.

Pathway	Gene	Product	Locus Tag
Myo-inositol, Rhizopine metabolism	*iol*E	myo-inosose-2 dehydratase	HWX41_RS07140
	*iol*G	myo-inositol 2-dehydrogenase	HWX41_RS07120
	*iol*D	3D-(3,5/4)-trihydroxycyclohexane-1,2-dione acylhydrolase (decyclizing)	HWX41_RS07135
	*iol*C	5-dehydro-2-deoxygluconokinase	HWX41_RS07125
	ND	5-deoxy-glucuronate isomerase	HWX41_RS07155
Putrescine and spermidine production and transport	*spe*B	agmatinase	HWX41_RS07085/HWX41_RS20910
	*spe*E	spermidine synthase	HWX41_RS20905
	ND	glutathionylspermidine synthase family protein	HWX41_RS10015/HWX41_RS16005
	*pot*D	spermidine/putrescine ABC transporter substrate-binding protein *Pot*D	HWX41_RS17570
	*pot*C	spermidine/putrescine ABC transporter permease *Pot*C	HWX41_RS17575
	*pot*B	spermidine/putrescine ABC transporter permease *Pot*B	HWX41_RS17580
	*pot*A	spermidine/putrescine ABC transporter ATP-binding protein *Pot*A	HWX41_RS17585
GABA biosynthesis	ND	gamma-glutamyl-gamma-aminobutyrate hydrolase family protein	HWX41_RS17805/HWX41_RS07225

Key: ND—not determined.

Table S12. Genes involved in lignin degradation and degradation against toxic peroxides.

Pathway	Gene	Product	Locus Tag
Lignin degradation	*ND*	multicopper oxidase domain-containing protein	HWX41_RS14790
Degradation of toxic peroxides	*ND*	Superoxide dismutase	HWX41_RS16575
	*sod*A	Superoxide dismutase	HWX41_RS20530/HWX41_RS02855
	*sod*A	superoxide dismutase family protein	HWX41_RS23120
	*ahp*C	Peroxiredoxin	HWX41_RS24440
	*kat*B	Catalase	HWX41_RS07625/HWX41_RS09520
	*kat*B	Catalase	HWX41_RS18175
	bcp	thioredoxin-dependent thiol peroxidase	HWX41_RS25065
	tpx	thiol peroxidase	HWX41_RS01185
	*ahp*C	Peroxiredoxin	HWX41_RS24440
	ND	organic hydroperoxide resistance protein	HWX41_RS01930
Metabolism of alcohols and aldehydes	*adh*P	alcohol dehydrogenase *Adh*P	HWX41_RS13135
	*adh*P	alcohol dehydrogenase *Adh*P	HWX41_RS13135
	ND	zinc-binding alcohol dehydrogenase family protein	HWX41_RS06915
	ND	iron-containing alcohol dehydrogenase	HWX41_RS05805/HWX41_RS13340
	*adh*E	bifunctional acetaldehyde-CoA/alcoholdehydrogenase	HWX41_RS02425

Key: ND—not determine.

Table S13. Genes involved in biological control.

Pathway	Gene	Product	Locus Tag
Volatile degradation	*aco*A	acetoin:2,6-dichlorophenolindophenol oxidoreductase subunit alpha	HWX41_RS10755
	*aco*B	acetoin:2,6-dichlorophenolindophenol oxidoreductase subunit beta	HWX41_RS10760
2,3-Butanediol	*ilvA*	threonine ammonia-lyase *ilv*A	HWX41_RS14970
	*ilv*D	dihydroxy-acid dehydratase	HWX41_RS14975
	*ilv*C	ketol-acid reductoisomerase	HWX41_RS16965
	*ilv*N	acetolactate synthase small subunit	HWX41_RS16970
	*ilv*B	acetolactate synthase large subunit	HWX41_RS16975
	*ilv*E	branched-chain-amino-acid transaminase	HWX41_RS16980
Isoprene	*isp*G	4-hydroxy-3-methylbut-2-enyl-diphosphate synthase	HWX41_RS02835
Colonization	*min*D	septum site-determining protein *Min*D	HWX41_RS02025

Table S14. Physical and chemical analysis of the soil samples.

Parameter	Result Output
Manganese (mg/kg)	43.57
Phosphorus (mg/kg)	74.93
Organic carbon (%)	1.19
Total nitrogen (%)	0.10
pH (H_2O)	7.40
Magnesium (mg/kg)	642.00
Organic matter (%)	3.20
Potassium (mg/kg)	399.00
Iron (mg/kg)	2.00
Clay (%)	22.20
Sand (%)	68.30
Silt (%)	7.92
